# Adequate antibiotic therapy prior to ICU admission in patients with severe sepsis and septic shock reduces hospital mortality

**DOI:** 10.1186/s13054-015-1000-z

**Published:** 2015-08-27

**Authors:** José Garnacho-Montero, Antonio Gutiérrez-Pizarraya, Ana Escoresca-Ortega, Esperanza Fernández-Delgado, José María López-Sánchez

**Affiliations:** Unidad Clínica de Cuidados Críticos, Hospital Universitario Virgen del Rocío/CSIC/Universidad de Sevilla, Avda. Manuel Siurot s/n, 41013 Sevilla, Spain; Instituto de Biomedicina de Sevilla (IBIS), Hospital Universitario Virgen del Rocío/CSIC/Universidad de Sevilla, Avda. Manuel Siurot s/n, 41013 Sevilla, Spain; Red Española de Investigación en Patología infecciosa (REIPI), Hospital Universitario Virgen del Rocío/CSIC/Universidad de Sevilla, Avda. Manuel Siurot s/n, 41013 Sevilla, Spain; Unidad Clínica de Enfermedades Infecciosas, Microbiología y Medicina Preventiva, Hospital Universitario Virgen del Rocío, Avda. Manuel Siurot s/n, 41013 Sevilla, Spain

## Abstract

**Introduction:**

In patients with severe sepsis and septic shock as cause of Intensive Care Unit (ICU) admission, we analyze the impact on mortality of adequate antimicrobial therapy initiated before ICU admission.

**Methods:**

We conducted a prospective observational study enrolling patients admitted to the ICU with severe sepsis or septic shock from January 2008 to September 2013. The primary end-point was in-hospital mortality. We considered two groups for comparisons: patients who received adequate antibiotic treatment before or after the admission to the ICU.

**Results:**

A total of 926 septic patients were admitted to ICU, and 638 (68.8%) had available microbiological isolation: 444 (69.6%) received adequate empirical antimicrobial treatment prior to ICU and 194 (30.4%) after admission. Global hospital mortality in patients that received treatment before ICU admission, between 0-6h ICU, 6–12h ICU, 12–24h ICU and after 24 hours since ICU admission were 31.3, 53.2, 57.1, 50 and 50.8% (*p*<0.001). The multivariate analysis showed that urinary focus (odds ratio (OR) 0.20; 0.09–0.42; *p*<0.001) and adequate treatment prior to ICU admission (OR 0.37; 0.24–0.56; *p*<0.001) were protective factors whereas APACHE II score (OR 1.10; 1.07–1.14; *p*<0.001), septic shock (OR 2.47; 1.57–3.87; *p*<0.001), respiratory source (OR 1.91; 1.12–3.21; *p*=0.016), cirrhosis (OR 3.74; 1.60–8.76; *p*=0.002) and malignancy (OR 1.65; 1.02–2.70; *p*=0.042) were variables independently associated with in-hospital mortality. Adequate treatment prior to ICU was a protective factor for mortality in patients with severe sepsis (n=236) or in septic shock (n=402).

**Conclusions:**

The administration of adequate antimicrobial therapy before ICU admission is decisive for the survival of patients with severe sepsis and septic shock. Our efforts should be directed to assure the correct administration antibiotics before ICU admission in patients with sepsis.

## Introduction

Acute management of patients with severe sepsis and septic shock is a very frequent task for ICU physicians. Initially, this management includes hemodynamic support and the administration of adequate antibiotics. Timely antibiotic administration is associated with decreased morbidity and mortality in critically ill patients with septic shock [1–3]. Treatment protocols aiming at the rapid administration of adequate antibiotics are now considered a key element for improving the outcome of patients with septic shock [4]. Although the survival benefit associated with prompt antibiotic administration is clearly established for patients with septic shock, controversial results have been reported in patients with severe sepsis without shock [3, 5].

Although sepsis may occur in patients previously admitted to the ICU for other reasons, the great majority of sepsis episodes are diagnosed in hospital wards or in the emergency department (ED) and from these areas are transferred to the ICU. Several observational studies agree that only 10–30 % of patients are located in the ICU when sepsis is identified [3, 6, 7]. It is noteworthy that acquisition of the infection before ICU admission has been identified as independently associated with early death in a large cohort of patients with sepsis [8].

The remaining episodes are diagnosed in the ED (50–60 %) and sepsis recognition is performed in other areas of the hospital (general wards or high-risk areas such as hematological or transplant units) in approximately 20–40 % of the cases [3, 6, 7]. The first antibiotic doses are therefore usually administered outside the ICU when culture results are not yet available [9].

Our aim is to analyze the impact on mortality of receiving adequate antimicrobial therapy before ICU admission compared with patients treated correctly after ICU admittance, in a cohort of patients admitted to the ICU for severe sepsis or septic shock. To better understand the burden of early adequate therapy on outcome, we performed this same analysis in severe sepsis and in septic shock separately.

## Materials and methods

This study was performed in the ICU of Hospital Virgen del Rocío, a 40-bed medico-surgical unit in a large university hospital, from 1 January 2008 to 30 September 2013. The Institutional Review Board of Virgen del Rocío Hospital approved this protocol, waiving the need for informed consent given the observational design of this study.

All adult patients with severe sepsis or septic shock on admission to the ICU were included in a prospectively collected database. A previous study has been published with records included in this database [10]. The study included all patients in whom antimicrobial therapy was adequate before and after ICU admission and those who received adequate antibiotics only after being admitted to the ICU.

Based on the recommendations of the Surviving Sepsis Campaign [11], the hospital has a protocol for the management of severe sepsis and septic shock that includes initial therapy outside the ICU (fluid resuscitation, vasoactive drugs, source control, and empirical antimicrobial therapy). The protocol for antimicrobial therapy is updated annually by the Committee for Surveillance of Infections and Antimicrobial Use of Hospital Virgen del Rocío and is available on the hospital intranet.

The following variables were recorded: gender, age, chronic organ insufficiencies as defined by the Acute Physiology and Chronic Health Evaluation (APACHE) II scale, and other comorbidities (alcoholism, smoking habit, diabetes mellitus, noncure malignancy, and previous surgery) as defined previously [12]. At admission to the ICU, severity of the illness was evaluated by the APACHE II score and by the Sequential Organ Failure Assessment (SOFA) scale that was also registered on admission and during the subsequent clinical course [13, 14].

In all patients, the following variables were also recorded: surgical or medical patient, nosocomial or community-acquired infection, clinical picture of sepsis (American College of Chest Physicians and the Society of Critical Care Medicine definitions), bacteremia, microbiologically documented infection, microorganism isolated, and appropriateness of empirical antibiotics. *Candida* infection was defined when it was recovered in blood culture or in any sterile sample. Isolation of *Candida* spp. in sputum or other respiratory samples was considered contamination. Bacteria included in the ESKAPE group (*Enterococcus faecium*, methicillin-resistant *Staphylococcus aureus*, *Klebsiella pneumoniae*, *Acinetobacter baumannii*, *Pseudomonas aeruginosa*, and *Enterobacter* species) which caused episodes were considered “difficult-to-treat” pathogens [15].

Antimicrobial therapy was considered adequate when the antibiotics prescribed covered all of the isolated pathogens (in blood and/or in the infection focus) and the dose and pattern of administration were in accordance with current standards. Patients were divided into two categories: patients in whom the antimicrobial therapy was adequate before ICU admission and those who received adequate antibiotics only after being admitted to the ICU.

Acquisition of a nosocomial infection was defined as the development of nosocomial pneumonia, primary bacteremia, and catheter-related bloodstream infection during the ICU stay following current definitions [16]. All patients were followed up until death or hospital discharge.

### Statistical analysis

Discrete variables were expressed as counts (percentages) and continuous variables as means ± standard deviation. The chi-square test or Fisher’s exact test was used for categorical variables, and the Mann–Whitney U test or Kruskal–Wallis test was used for continuous variables. To identify independent variables associated with in-hospital mortality, as well as assessing the impact of adequate antimicrobial therapy before ICU admission on the prognosis, we performed multivariate analysis by a binomial logistic regression model. To avoid spurious associations, variables entered into the regression models were those with a relationship in univariate analysis (*p* <0.05), those with a plausible relationship with the dependent variable, or those that were clinically significant. Results are presented as odds ratio (OR) and 95% confidence interval (CI). Potential explanatory variables were checked for colinearity before inclusion in the regression models using the tolerance and variance inflation factor. The threshold for statistical significance was defined as *p* <0.05. Data analysis was performed using SPSS for Windows 19.0 (SPSS, Inc., Chicago, IL, USA).

## Results

The total cohort comprised 926 consecutive patients who were admitted to the ICU with the diagnosis of severe sepsis or septic shock. Sepsis was acquired in the community in 66.9 % of the cases and an infection acquired in hospital wards was the cause of sepsis in 33.1 % of the episodes. The median time elapsed from triage in the ED to ICU admission was 5 hours.

Microbiological documentation was obtained in 641 cases (69.2 %). Three patients received adequate antimicrobial therapy before ICU admission but the regimen chosen once the patient was in the ICU was not correct. These patients were excluded; therefore 638 patients constitute the study cohort (402 with septic shock and 236 with severe sepsis). The median time elapsed from sepsis identification to the administration of adequate antimicrobial therapy was 4 hours.

The comparison of patients treated adequately before ICU admission and those who received adequate antimicrobial therapy once they were in the ICU is presented in Table 1. Inadequate therapy before ICU admission was prescribed more frequently in nosocomial sepsis than in community-acquired episodes (44.7 vs. 23 %; *p* <0.001). Notably, severity of illness at admission to the ICU was similar in both groups of patients. A higher proportion of episodes caused by difficult-to-treat pathogens was found in patients who received adequate antibiotic only after being in the ICU. As expected, difficult-to-treat pathogens were more frequently isolated in nosocomial infections than in community-acquired episodes (36.6 vs. 16.6 %; *p* <0.001).

**Table 1:** Features of patients with adequate empirical antimicrobial therapy before or after ICU admission

	Before ICU (%)	Post ICU (%)	*p* value
	(*n* = 444)	(*n* = 194)	
Age	61 (47–71)	66 (53–73)	0.004
Gender (female)	200 (45)	72 (36.9)	0.056
APACHE II score	18 (12–23)	19 (14–24)	0.149
SOFA (median, IQR)	7 (4–10)	8 (5–10)	0.170
Worst SOFA	8 (5–12)	9 (6–12)	0.044
Surgical admission	225 (50.6)	115 (59)	0.060
Acquisition			
Community acquisition	324 (73)	97 (50)	<0.001
Hospital acquisition	120 (27)	97 (50)	
Comorbidities			
Diabetes	102 (23)	46 (23.6)	0.865
COPD	34 (7.6)	26 (13.3)	0.024
Cirrhosis	24 (5.4)	16 (8.2)	0.183
Malignancy	75 (16.9)	46 (23.6)	0.047
Chronic renal failure	38 (8.6)	11 (5.6)	0.202
Immunosuppression	68 (15.3)	33 (16.9)	0.608
Heart failure	24 (5.4)	9 (4.6)	0.678
Sepsis source			
Chest	80 (18)	29 (14.9)	0.330
Urinary	59 (13.3)	16 (8.2)	0.066
Abdomen	(45)	106 (54.4)	0.030
Central nervous system	18 (4.1)	2 (1)	0.043
Soft tissue	51 (11.5)	19 (9.7)	0.516
Catheter	15 (3.4)	8 (4.1)	0.651
Unidentified	12 (2.7)	8 (4.1)	0.349
Positive blood culture	224 (50.5)	97 (49.7)	0.869
Type of pathogen isolated			
Gram-positive	111 (25)	36 (18.6)	0.080
Gram-negative	196 (44.1)	88 (45.4)	0.847
Anaerobic	7 (1.6)	1 (0.5)	0.289
Fungus	5 (1.1)	8 (4.1)	0.028
Polymicrobial	120 (27)	59 (30.4)	0.304
Others	5 (1.1)	2 (1)	0.141
Difficult-to-treat pathogens	86 (19.3)	67 (34.3)	<0.001
Septic shock	281 (63.3)	121 (62.4)	0.825
Mechanical ventilation	172 (38.7)	100 (51.5)	0.002
Nosocomial infection	89 (20.1)	43/194 (22.2)	0.580
ICU stay	8 (5–15)	9 (5–17)	0.114
Hospital stay	25 (15–41)	38 (21–52)	0.052
ICU mortality	121 (27.2)	88 (45.4)	<0.001
Hospital mortality	139 (31.3)	103 (52.8)	<0.001

*APACHE* Acute Physiology and Chronic Health Evaluation, *COPD* chronic obstructive pulmonary disease, *CRBI* catheter-related bloodstream infection, *IQR* interquartile range, *SOFA* Sequential Organ Failure Assessment

Two-hundred and fourteen patients who were admitted from a general ward received the first dose of antibiotic in these wards (mainly in the internal medicine and general surgery wards). Otherwise, 390 patients with community-acquired infection received the first antibiotic in the ED and 23 patients received the first antibiotic in the operating room. One hundred and ninety-four patients were treated inadequately before ICU admission. Only 11 of them had not received any antimicrobial agent before ICU admission. The remaining patients (*n* = 183) had received antibiotic treatment but it was judged inadequate based on culture results received once the patient was in the ICU. The uncovered pathogens (*n* = 189) in these 183 patients are presented in Table 2 (in six patients, two pathogens not initially covered were isolated).

**Table 2:** Pathogens isolated in patients with inadequate empirical therapy before ICU admission

	Focus	Bloodstream
Gram-negative		
*Escherichia coli*	27 (26.5)	30 (34.5)
*Pseudomonas aeruginosa*	15 (14.7)	6 (6.9)
*Klebsiella pneumoniae*	11 (10.8)	9 (10.3)
*Acinetobacter baumannii*	7 (6.9)	4 (4.6)
*Proteus mirabilis*	2 (2)	1 (1.1)
*Enterobacter* spp.	5 (4.9)	4 (4.6)
Gram-positive		
Methicillin-resistant *Staphylococcus aureus*	2 (2)	5 (2.4)
*Enterococcus* spp.	15 (14.7)	6 (6.9)
*Staphylococcus* spp.	–	3 (3.4)
Fungi		
*Candida* spp.	8 (7.8)	11 (12.6)
Others	10 (9.8)	8 (9.2)
Total	102	87

**Fig. 1:**
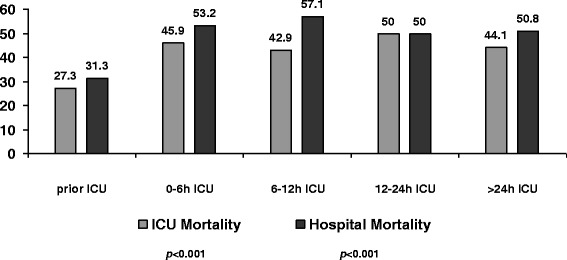
ICU and hospital mortality rates for the time range of adequate empirical antimicrobial therapy

Bivariate analysis of risk factors associated with hospital mortality is depicted in Table 3. Factors associated with fatality by multivariate analysis were APACHE II score, cirrhosis, malignancy, pulmonary source, and septic shock, whereas urologic origin and adequate antimicrobial therapy before ICU admission were protective factors (Table 3). As shown in Fig. 1, ICU and hospital mortality rates were significantly lower in patients treated adequately before ICU admission but without difference regarding whether the adequate therapy was initiated in the first 6 hours in the ICU, between 6 and 12 hours, between 12 and 24 hours, or after more than 24 hours in the ICU (*p* <0.001).

**Table 3:** Bivariate and multivariate analyses of risk factors associated with hospital mortality

	Alive (%) (*n* = 397)	Death (%) (*n* = 241)	*p* value	Unadjusted OR (95% CI)	*p* value	Adjusted OR (95% CI)	*p* value
Age	60 (45–71)	65 (55–73)	<0.001	1.01 (1.00–1.25)	0.050		
Female gender	176 (44.2)	96 (39.8)	0.248	0.81 (0.54–1.21)	0.314		
APACHE II score	16 (11–20)	22 (17–27)	<0.001	1.10 (1.07–1.14)	<0.001	1.10 (1.07–1.14)	<0.001
SOFA (median, IQR)	6 (4–9)	10 (7–12)	<0.001				
Worst SOFA	7 (4–10)	11 (9–14)	<0.001				
Surgical admission	213 (53.6)	129 (53.5)	0.975				
Hospital acquisition	133 (33.5)	84 (34.9)	0.662				
Comorbidities							
Diabetes	82 (20.6)	66 (27.4)	0.054				
COPD	30 (7.6)	30 (12.4)	0.040	1.10 (0.57–2.12)	0.755		
Cirrhosis	10 (2.5)	30 (12.4)	<0.001	3.52 (1.49–8.28)	0.004	3.74 (1.60–8.76)	0.002
Malignancy	60 (15.1)	61 (25.3)	0.002	1.50 (0.90–2.48)	0.112	1.65 (1.02–2.70)	0.042
Chronic renal failure	26 (6.5)	23 (9.5)	0.173				
Immunosuppression	47 (11.8)	54 (22.4)	<0.001	1.47 (1.85–2.54)	0.168		
Heart failure	18 (4.5)	15 (6.2)	0.350				
Sepsis source							
Chest	55 (13.8)	54 (22.4)	0.006	1.84 (1.07–3.15)	0.026	1.91 (1.12–3.21)	0.016
Urinary	64 (16.1)	11 (4.5)	<0.001	0.20 (0.09–0.42)	<0.001	0.20 (0.09–0.42)	<0.001
Abdomen	188 (47.3)	118 (48.9)	0.730				
Central nervous system	15 (3.7)	5 (2.1)	0.228				
Soft tissue	39 (9.8)	31 (12.8)	0.241				
Catheter	15 (3.7)	8 (3.3)	0.756				
Unidentified	11 (2.7)	9 (3.7)	0.504				
Difficult-to-treat pathogens	94 (23.6)	59 (24.5)	0.859				
Positive blood culture	196 (49.3)	125 (51.9)	0.576				
Septic shock	208 (52.3)	194 (80.5)	<0.001	2.55 (1.62–4.03)	<0.001	2.47 (1.57–3.87)	<0.001
Nosocomial infection	73 (18.3)	58 (24.1)	0.070				
Prior ICU adequate antimicrobial therapy	305 (76.8)	139 (57.7)	<0.001	0.37 (0.25–0.57)	<0.001	0.37 (0.24–0.56)	<0.001

*APACHE* Acute Physiology and Chronic Health Evaluation, *CI* confidence interval, *COPD* chronic obstructive pulmonary disease, *IQR* interquartile range, *OR* odds ratio, *SOFA* Sequential Organ Failure Assessment

Four hundred and two patients presented septic shock at admission to the ICU. The mortality rate was 48.3 %. The bivariate analysis revealed that female gender, APACHE II, SOFA, cirrhosis, malignancy, and chest source of sepsis were significantly higher in nonsurvivors whereas a urinary source of sepsis and adequate antimicrobial therapy prior to admission to the ICU were more frequently administered in survivors than in nonsurvivors (79.4 vs. 60 %; *p* <0.001). In the multivariate analysis (Table 4), only four of these variables were independently associated with in-hospital mortality. Of note, adequate antimicrobial therapy before ICU admission was a protective factor for mortality (OR 0.40, 95% CI 0.24–0.65; *p* <0.001).

**Table 4:** Multivariate analysis of risk factors for hospital mortality in patients with severe sepsis and septic shock

	Severe sepsis		Septic shock	
	Adjusted OR (95% CI)	*p* value	Adjusted OR (95% CI)	*p* value
Age	1.02 (1.00–1.05)	0.033		
APACHE II score	1.07 (1.01–1.14)	0.020	1.11 (1.07–1.15)	<0.001
Cirrhosis			4.49 (1.55–13.04)	0.006
Immunosuppression	4.39 (1.64–11.72)	0.003		
Urinary sepsis source			0.11 (0.05–0.27)	<0.001
Nosocomial infection	6.53 (2.74–15.55)	<0.001		
Prior ICU adequate antimicrobial therapy	0.29 (0.13–0.63)	0.002	0.40 (0.24–0.65)	<0.001

*APACHE* Acute Physiology and Chronic Health Evaluation, *CI* confidence interval, *OR* odds ratio

In the subgroup of patients with severe sepsis at admission to the ICU, 47 patients (19.9 %) died during hospitalization. Development of septic shock occurred less frequently in patients with adequate antimicrobial therapy prior to admission to the ICU than in those patients who received adequate antimicrobial therapy only after being admitted to the ICU (9.5 vs. 18 %; *p* = 0.002). The bivariate analysis revealed that age, APACHE II score, SOFA, chronic obstructive pulmonary disease (COPD), presence of immunosuppression, history of chronic heart failure, and development of nosocomial infection were significantly higher in nonsurvivors whereas adequate antimicrobial therapy prior to admission to the ICU was more frequently administered in survivors than in nonsurvivors (74.5 vs. 48.9 %; *p* <0.001). In the multivariate analysis, only five of these variables (Table 4) were independently associated with in-hospital mortality. Again, adequate antimicrobial therapy before ICU admission was included in the final regression model (OR 0.29, 95% CI 0.13–0.63; *p* = 0.002).

## Discussion

The present study demonstrates that, in septic patients, the administration of adequate empirical antimicrobial therapy before ICU admission is a protective factor for mortality after adjusting for numerous confounders. Importantly, mortality rates were significantly lower in patients treated adequately before ICU admission compared with those received adequate therapy even in the first 6 hours in the ICU. This advantage in terms of survival is present in patients with severe sepsis or in patients with septic shock.

Our model for in-hospital mortality includes, apart from the use of adequate antimicrobial therapy before ICU admission, variables frequently identified as predictors of fatality in large cohorts of sepsis, such as severity of illness, certain comorbidities, and the source of infection. Although the origin of sepsis has not been identified as a determinant of mortality [17], others have concluded that an urological source of sepsis is a protective factor and pneumonia is associated with a higher risk of death [18, 19].

Various studies have demonstrated the strong relationship existing between the delay in the administration of the first dose of antibiotics and mortality in patients with septic shock [1, 2]. The rate of inadequate empirical therapy in critically ill patients with sepsis depends primarily on the incidence of multidrug resistance pathogens and the adhesion to clinical guidelines, and ranges from 10 to almost 40 % [18, 20, 21]. In the present series, the great majority of the patients (98.3 %) had received at least one dose of antimicrobial before ICU admission although it was inadequate in 31 % of them.

Emergency room utilization has increased over the last decade worldwide. The admission rate for sepsis from the ED has increased in comparison with admissions from hospital wards [22]. Outcomes for critically ill patients are influenced by whether or not optimal intensive support is delivered in a timely manner. Up to 20 % of the patients with severe sepsis or septic shock do not fulfill diagnostic criteria in the first 3 hours in the ED [23, 24]. In fact, sepsis is the predominant diagnostic category in patients with delayed transfer from the ED to the ICU [25, 26].

Diverse studies have reported a significant delay in the administration of appropriate antibiotics in patients with severe sepsis and septic shock admitted to the ICU from the ED [27, 28]. Delays were greater for those in whom the diagnosis of sepsis was not considered initially. Sepsis can be difficult to diagnose because its clinical presentation mimics the symptoms of several other critical care conditions [29]. This fact may, at least in part, explain that only 60–80 % of the patients received antibiotics in the ED in two recent retrospective studies carried out in the USA [30, 31].

In a small unicenter study, correct antibiotic coverage was prescribed by emergency physicians in 82 % of the patients with culture-positive sepsis [9]. This was a retrospective study that found no impact on mortality considering the time elapsed from triage to the first dose of antibiotics in the ED. In contrast, a delay in the administration of appropriate antimicrobials from triage was independently associated with mortality. The use of broad-spectrum antibiotics was considered adequate in culture-negative episodes [32].

Antimicrobial resistance is an alarming clinical problem that is recognized internationally as one of the largest threats to human health. As in other series [9, 20], we observed that Gram-negative organisms comprise the most frequent cause for errors in empiric coverage. When antimicrobial therapy is initiated in the ED, multidrug-resistant bacteria are covered very infrequently [30].

Approximately one-third of patients included in our series were transferred from the general ward to the ICU with a diagnosis of sepsis. Clinically significant delays in the process of care, including the administration of appropriate antibiotics, have been observed in patients with severe sepsis as it is in patients with septic shock in the general wards compared with those who presented septic shock when already admitted to the ICU [33]. In medical and surgical patients admitted to the general wards of a university-affiliated hospital, the median interval from the onset of severe sepsis or septic shock to the administration of antibiotic was 4 hours [34].

The impact on mortality of delayed antimicrobial therapy is not clearly established in patients with severe sepsis as it is in patients with septic shock [1, 2, 5]. Recently, a statically significant increment in the probability of death associated with the number of hours of delay for the first antibiotic administration was demonstrated in a large cohort of patients with severe sepsis and septic shock, although the subgroup of patients without shock was not evaluated specifically [3]. In a prospective study carried out in the EDs of three large hospitals, an increase in mortality with each hour delay to administration of antibiotics was demonstrated in patients with shock but not in patients with severe sepsis without development of shock [35]. In patients with community-acquired severe sepsis, a 6-hour delay or more in administration of the initial dose of antimicrobial was identified as an independent predictor of mortality and the authors could not demonstrate an independent impact on mortality during the previous hours [36].

We also found that progression to septic shock occurred more frequently in patients with severe sepsis who received inadequate antimicrobial therapy prior to ICU admission. Conversely, in hemodynamically stable patients presenting to the ED with sepsis, the use of appropriate antibiotics was similar in patients who developed shock within 72 hours and those who remained hemodynamically stable [35]. We and others have previously demonstrated that inadequate antimicrobial therapy is associated with a higher degree of organ dysfunction in the ICU (including cardiovascular system) than those who received adequate antimicrobial therapy [21, 37].

There are several limitations to this study. Management of septic patients involves infection control and organ support. Control of infection is achieved through prompt administration of adequate antibiotics and surgical drainage when necessary. We did not monitor timing of fluid resuscitation or source control. Prompt and aggressive fluid resuscitation is associated with a higher survival rate in severe sepsis and septic shock [38]. Unfortunately, our study design did not include registering the time lag between the detection of the first signs or symptoms of sepsis and the administration of the first antibiotic dose, which hinders comparisons with series that analyzed the delay of administration of the first antibiotic dose.

## Conclusions

We have demonstrated that the initiation of effective antimicrobial therapy before ICU admission is a critical determinant of outcome in patients with severe sepsis and septic shock. Our data highlight that the administration of adequate antibiotics once the patient is in the ICU is not sufficient because the mortality rate is significantly higher although the initiation of the correct antimicrobial therapy takes place in the first hours after ICU admission. Renewed efforts should be implemented to minimize the prescription of incorrect antimicrobials in septic patients, especially outside the ICU.

## Key messages

- Approximately, one-third of the patients included in our series were transferred from the general ward to the ICU with a diagnosis of sepsis.
- The progression to septic shock occurred more frequently in patients with severe sepsis who received inadequate antimicrobial therapy prior to ICU admission.
- In septic patients, the administration of adequate empirical antimicrobial therapy before ICU admission is a protective factor of mortality.

## Abbreviations

APACHE: Acute Physiology and Chronic Health Evaluation
CI: Confidence interval
COPD: Chronic obstructive pulmonary disease
ED: Emergency department
OR: Odds ratio
SOFA: Sequential Organ Failure Assessment.
